# Workplace Sedentary Behavior and Productivity: A Cross-Sectional Study

**DOI:** 10.3390/ijerph17186535

**Published:** 2020-09-08

**Authors:** Sara K. Rosenkranz, Emily L. Mailey, Emily Umansky, Richard R. Rosenkranz, Elizabeth Ablah

**Affiliations:** 1 Department of Food, Nutrition, Dietetics and Health, Kansas State University, 1105 Sunset Ave, Rm 322, Manhattan, KS 66502, USA; Ricardo@ksu.edu; 2 Department of Kinesiology, Kansas State University, 8 Natatorium, Manhattan, KS 66506, USA; Emailey@ksu.edu; 3 Department of Population Health, University of Kansas School of Medicine–Wichita, 1010 N Kansas, Wichita, KS 67214, USA; Emily.Umansky@gmail.com (E.U.); Eablah@kumc.edu (E.A.)

**Keywords:** sitting, fatigue, job satisfaction, worksite, office-based, government, employees, employers

## Abstract

Reducing sedentary behavior in the workplace has become an important public health priority; however, some employers have expressed concerns regarding the potential for reduced productivity if employees are not seated while at work. Therefore, the aim of this study was to determine the relationship between workplace sedentary behavior (sitting time) and work productivity among full-time office-based employees, and further to investigate other potential factors associated with productivity. A 19-item online self-report survey was completed by 2068 government employees in Kansas. The survey assessed workplace sedentary behavior, work productivity, job satisfaction, and fatigue. Overall, office workers reported high levels of sedentary time (mean > 78%). The primary results indicated that sitting time was not significantly associated with productivity (*β* = 0.013, *p* = 0.519), but job satisfaction and fatigue were positively (*β* = 0.473, *p* < 0.001) and negatively (*β* = −0.047, *p* = 0.023) associated with productivity, respectively. Furthermore, participants with the highest level of sitting time (>91% of the time) reported lower job satisfaction and greater fatigue as compared with the lowest level of sitting time (<75% of the time). Taken together, these results offer promising support that less sitting time is associated with positive outcomes that do not seem to come at the expense of productivity.

## 1. Introduction

High levels of sedentary behavior are commonplace in the United States. Researchers estimate that adults are sedentary for approximately 7.7 h each day [1]. Desk jobs, television watching, and commuting to work each increase the amount of sedentary time for adults [2,3]. Sedentary behavior is especially common in the workplace; office workers sit for about 70% of the time spent at work [4,5,6].

There is now a wealth of evidence that demonstrates a relationship between increased sedentary behavior and a higher risk of a host of negative health outcomes, including cardiovascular disease, type II diabetes, obesity, several types of cancer, musculoskeletal conditions, and all-cause mortality [7,8,9]. In addition to physical health outcomes, increased sedentary behavior is also associated with an increased risk of depression [10]. In light of the detrimental health effects of high levels of sedentary behavior, public health researchers have committed considerable efforts toward interventions designed to reduce sedentary behavior, particularly among office workers. However, ensuring that reductions in sedentary behavior will not damage productivity is likely to be important for employer buy-in, particularly given that employees in focus groups have voiced concerns about the potential for decreased productivity in the wake of interventions targeting sedentary behavior in the workplace [11,12]. Workplace health practitioners have also indicated productivity concerns as the key barrier to making changes in the workplace [13]. These concerns seem logical, given that reductions in sedentary behavior may take employees away from their workstations, or conceivably could replace work time with physical activity time.

Productivity measures are relatively common in worksite-based, sedentary behavior studies; reviews have identified 20 studies that include some measure of productivity [14,15,16]. Some worksite-based intervention studies that addressed sedentary behavior have shown no effect on productivity [17,18], while others suggest an increase or perceived increase in productivity [19,20]. Reviews of this body of evidence suggest that, overall, interventions to reduce sedentary behavior do not significantly impact productivity. However, this body of research also suggests that additional investigation of the relationship between workplace sedentary behavior and productivity is warranted as the association between these two variables has not been consistent [14,15,16].

The results from a cross-sectional study conducted by Munir and colleagues (2015) suggested that greater sitting time is associated with lower work engagement [21]. Two additional cross-sectional studies have examined the relationship between workplace sitting time and productivity, with contrasting results. Puig-Ribera and colleagues (2015) determined that there was not a significant association between workplace sitting time and productivity among a sample of approximately 550 office employees at a Spanish university [22]. In contrast, a study of approximately 2500 Japanese adults by Ishii et al. (2018) suggested that employees aged 20 to 39 y who had higher job-related sedentary behavior were more likely to report being less efficient than those with low job-related sedentary behavior [23]. Given these seemingly contradictory results, further research that incorporates additional factors that may be associated with sedentary behavior and/or productivity—such as job satisfaction and fatigue—is warranted.

The connection between job satisfaction and productivity is one that researchers have examined for more than 60 years [24]. A review from 1985 estimated that the overall correlation between job satisfaction and productivity is small (*r* = 0.17), whereas a later review suggested a stronger overall association (*r* = 0.3) [25,26]. As compared with the association with productivity, there is little research on the topic of the association between sedentary behavior and job satisfaction. Gorman and colleagues’ intervention to reduce sedentary behavior showed a small, non-significant improvement in job satisfaction [27]. Outside of this particular study, thus far, job satisfaction has not been an outcome of interest in investigations of sitting time. Additional research that examines sedentary behavior and job satisfaction could elucidate a potential relationship between these two variables.

High levels of fatigue appear to be associated with low productivity, and this relationship has been shown across various measures of productivity. One study that used a national cross-sectional telephone survey indicated that workers who report fatigue are more likely to report health-related “lost productive time” in comparison with those who do not report fatigue [28]. Others report that increased fatigue is also correlated with reduced perceived performance and reduced work productivity [29,30]. Furthermore, some research shows that interventions for reducing sitting time also reduce fatigue [31,32]. Altogether, previous studies indicate that job satisfaction and fatigue might be important factors that could help to explain a potential relationship between workplace sedentary behavior and productivity.

Though researchers have previously included productivity measures in intervention studies designed to reduce workplace sedentary behavior, the relationship between these two factors is not fully understood. Further, analysis of this relationship with statistical adjustment for important factors, such as job satisfaction and fatigue, which also may be associated with productivity, is necessary. Previous research indicates significant employer concern regarding loss of productivity with decreased sedentary time. In order to address the concern of employers in a sector of the workforce likely to encounter barriers to changes in workplace policies, the primary purpose of this study was to investigate the relationship between workplace sedentary behavior and productivity among a large, representative sample of full-time government office workers in the State of Kansas.

## 2. Materials and Methods

### 2.1. Participants

The Human Subjects Research Institutional Review Board at Kansas State University (IRB# 8886) approved this cross-sectional study. Eligible participants for this study were full-time, adult employees of 12 of the largest state government agencies in Kansas. These individuals were identified as potentially eligible participants for a longitudinal intervention study on sedentary behavior in the workplace. A Kansas State government human resources official sent an invitation to complete an interest survey to the work emails of all employees. The study consisted of a single electronic survey that could be completed at any computer with internet access. The study occurred over a fifteen-day period in March 2018. Participants did not receive compensation for their participation in the study.

### 2.2. Methods

The 19 survey items (see Appendix A for the full survey) for the current study were designed to measure five variables, using self-report responses: demographics (eight items), sedentary behavior (one item), productivity (three items), job satisfaction (four items), and fatigue (three items).

Basic demographic data on gender, age, ethnicity, race, marital status, and education level were collected from participants, as were data about the name of their employer and whether they worked 30 hours or more per week. Sedentary behavior was self-reported as the percentage of time during an average workday in the past week they spent sitting [33]. To assess productivity, job satisfaction, and fatigue, survey items from existing validated questionnaires were used [34,35]. All measures of sedentary behavior, productivity, job satisfaction, and fatigue prompted participants to consider the past seven days when responding. In the interest of keeping the survey at a manageable length, the instrument for this study used subsections of the original validated measures. Sample items are displayed in Table 1.

### 2.3. Data Analyses

The data were analyzed using IBM SPSS Statistics for Windows, Version 25.0. Armonk, NY: IBM Corp. Descriptive statistics for each construct are presented as mean ± *SD*. Significance was set as *p* < 0.05. For the productivity (three items, α = 0.88) and job satisfaction (four items, α = 0.84) subscales, an aggregate score was created by calculating the mean of the responses within each subscale. Data from participants who completed more than half of the items within the subscale (two of three items for productivity or three of four items for job satisfaction) were used for analyses involving each scale. When an item within a subscale was incomplete (*n* = 145/14,464 total items), the missing score was replaced by the average score of the entire participant sample for that item. The three fatigue items were multiplied together to create an overall fatigue index rather than an average score, which would not have been appropriate given the disparate structure of scale items. Missing data points were not replaced for any of the fatigue index items.

Parametric assumptions were checked for each variable and the sitting time item was logarithmically transformed and reflected to correct for skewness and kurtosis. Following checks for collinearity, a three-step linear regression analysis (entry method) was used to determine the association between sitting time and productivity, statistically adjusting for potential confounding variables in steps 2 and 3. At step 2, job satisfaction and fatigue index were entered into the regression model, and at step 3, demographic factors (race, age, gender, education) were entered. Changes in R^2^ were determined at each step. Following linear regression, additional analyses were conducted to determine differences in productivity, job satisfaction, and fatigue index by three levels (lowest, middle, and highest) of percentage of time spent sitting during a typical workday. Three levels were used specifically to deal with non-normal distribution, potentially non-linear relationships, and to contrast the lowest and highest levels while minimizing misclassification. Owing to the kurtosis for the sitting time variable and the violation of additional parametric assumptions for the fatigue index score, two-sided asymptotic Kruskal–Wallis tests of independent samples with Bonferroni corrections were used to assess differences in productivity, job satisfaction, and fatigue index, comparing the highest level to the lowest level for percentage sitting time for a typical work day.

## 3. Results

### 3.1. Participants

A total of 2629 participants across 12 government agencies began the study by completing the interest survey. Data from participants who did not answer any of the job satisfaction, fatigue, and productivity measures (*n* = 367) and from participants who were not full-time employees (*n* = 193) were removed. After excluding one additional participant who reported being younger than 18 years old, 2068 participants remained in the final dataset.

The data from the job satisfaction scale were excluded from analyses for three participants who had missing data for two of the four items. In cases where participants answered all but one item within a subscale (productivity or job satisfaction), the missing value was replaced. The number of missing values for each item ranged from 3 to 62, or from 0.14% to 3.00% of the data for a single item (see Appendix B).

### 3.2. Demographics

Participant demographics are displayed in Table 2. The average age of participants was 46 years (*SD* = 11.9 y). The sample of government employees was primarily female, with minimal racial or ethnic diversity. A majority (61%) reported being married, as compared with being widowed, divorced, separated, or single. A large majority of participants reported having completed at least some college; participants were most likely to report having completed a bachelor’s degree.

The mean (*SD*) for each of the 19 survey items is reported in Table 3. Participants reported sitting for an average of >78% of their work day. For all measures of productivity, job satisfaction, and fatigue, higher numbers indicate a greater level (e.g., a higher level of efficiency).

### 3.3. Associations between Sedentary Behavior and Productivity

Table 4 displays the results from the three-step linear regression analyses. At step one, there was not a significant association between percentage time spent sitting during a typical work day and productivity. This association remained non-significant at step two as well as in the fully adjusted model (step three). At step two and step three, the addition of job satisfaction, fatigue index, and demographic variables significantly increased the explained variance in productivity within the model. Job satisfaction was positively associated with productivity within the partially and fully adjusted models, whereas fatigue index was negatively associated with productivity; however, this association was very small and would not be significant after correction for multiple comparisons. Age, gender, and education level were all significantly associated with productivity in the fully adjusted model; however, associations were very small.

### 3.4. Differences in Productivity, Job Satisfaction, and Fatigue Index by Level of Sitting Time

Kruskal–Wallis analyses were performed for productivity, job satisfaction, and fatigue index by level of percentage of time spent sitting during a typical work day. The omnibus tests for productivity (*p* = 0.011), job satisfaction (*p* < 0.001), and fatigue index (*p* < 0.001) were all significant. Further analyses examined the differences between the lowest level (0–74% time spent sitting; *n* = 560) and the highest level (91–100% time spent sitting; *n* = 420). Overall, productivity differed significantly according to the level of time spent sitting during a typical work day in the last 7 days (*H* = 8.940, *df* = 2, *p* = 0.011). Office workers who reported spending less than 75% of their work day sitting reported higher productivity (median = 8.0, interquartile range (IQR) = 7.0, 8.7) as compared with those who reported spending 91% or more of their typical work day sitting (median = 7.7, IQR = 6.7, 8.3; *H* = 2.184, *df* = 1, *p* = 0.029). After Bonferroni correction, however, the *p*-value no longer indicated that this difference was significant (*p_adj_* = 0.087).

Job satisfaction was also different by the level of percentage of time spent sitting (*H* = 33.471, *df* = 2, *p* < 0.001). Office workers in the lowest level for sitting reported higher job satisfaction (median = 7.0, IQR = 5.5, 8.0) as compared with the highest level (median 6.3, IQR = 4.8, 7.5; *H* = 4.750, *df* = 1, *p* <0.001). Finally, fatigue index differed significantly by the level of percentage of time spent sitting (*H* = 29.294, *df* = 1, *p* < 0.001). Participants in the lowest level of time spent sitting reported lower fatigue overall (median 84.0, IQR = 24, 224) as compared with those in the highest level (median 135.0, IQR = 42, 333; *H* = −4.334, *df* = 1, *p* < 0.001).

## 4. Discussion

The aim of this study was to investigate the relationship between workplace sedentary behavior and productivity in a large sample of office workers within government agencies in Kansas. Further, we sought to examine other important factors that may be associated with time spent sitting, or may help to explain the association, or lack thereof, between time spent sitting and productivity, including job satisfaction and fatigue. The primary results of this study indicated that there was not a significant association between workplace sedentary behavior and productivity among this sample of full-time government office workers when using self-report measures. Additionally, office workers in the lowest level of time spent sitting reported higher job satisfaction and lower fatigue as compared with those in the highest level of time spent sitting. Because previous research suggests that productivity concerns are a key barrier to implementing changes in the workplace that may decrease sedentary behavior, results from the current study may be interpreted positively with respect to alleviating employer concerns related to reducing sedentary behavior for their employees [11,12,13].

Overall, the results suggest that office workers in the current study were primarily sedentary during the workday, sitting for approximately 78% of their time spent at work. This percentage is near the upper edge of the range of 65–75% that previous studies of office-based sedentary behavior have demonstrated [4,5,6,36]. These results are generally consistent with previous findings, and reinforce the consensus that sedentary behavior is prevalent in office settings.

Our study’s results are similar to those found by Puig-Ribera and colleagues, which also indicated no relationship between these variables among employees at a Spanish university [22]. Collectively, these studies may help to explain why workplace interventions that reduce sedentary behavior do not typically result in changes in productivity [14,15,16]. Moreover, these data may suggest that researchers as well as employers should not expect changes in productivity as a result of interventions that effectively reduce workplace sedentary behavior.

Furthermore, when considered alongside the positive results with regard to better job satisfaction and lower fatigue in employees with lower levels of sitting time, the current results may actually encourage employers to consider implementing policies that aim to reduce sedentary behavior. Sitting time was not associated with productivity. Additionally, although the follow-on analyses indicated that office workers who reported sitting for less than 75% of their work day did report higher productivity, as compared with those who reported more than 91% of their day was spent sitting, the difference was not statistically significant, and likely not meaningful. Studies show that the use of a sit-to-stand desk or an active workstation has the potential to improve productivity overall—rather than simply not harming it when intervening in the work place—potentially through reductions in worker fatigue or discomfort [15,16]. Overall, the current results, along with previous research, suggest that reducing workplace sedentary behavior is unlikely to be associated with significant changes in productivity.

This study afforded the opportunity to examine potential associations between other factors that might impact productivity. Job satisfaction was positively associated with productivity in the partially adjusted (β = 0.481) as well as fully adjusted (β = 0.473) model, in agreement with previous research demonstrating a positive association between these variables [26,34]. In the follow-on analyses in the current study, which reflected unadjusted associations between sedentary time and job satisfaction, job satisfaction was higher in the office workers who reported the lowest level of time spent sitting as compared with those who reported the highest level of time spent sitting. This finding adds support to the fully adjusted regression model, indicating that job satisfaction warrants further investigation in future studies, particularly intervention studies, to determine whether reductions in sedentary time could contribute to improved job satisfaction. There have been a few intervention studies that have examined the associations between sedentary behavior in the workplace and job satisfaction. However, one cross-sectional study conducted in sedentary employees who sat at least eight hours per day showed that employees who did not participate in regular physical activity had lower job satisfaction and reported a poorer quality of life, as compared with employees who obtained at least one hour of physical activity at least three days per week [37]. A natural experiment where activity-based working (ABW) principles were used in one workplace showed no significant effect on overall job satisfaction as compared with the comparison workplace [38]. However, study authors reported that productivity was slightly reduced in the ABW workplace, as compared with the comparison workplace. Another cross-sectional study indicated that standing or walking work that was not strenuous, as compared with sedentary work, was associated with higher odds for reporting being “very satisfied” with the job [39]. The opposite relationship was reported for heavy or strenuous work, and overall, the strongest predictors for job satisfaction were social support from colleagues and superiors, as well as influence at work.

One other notable finding from the current study was that the calculated fatigue index score was negatively associated with productivity, albeit a very weak association, indicating that office workers who reported greater levels of fatigue also reported lower levels of productivity. This finding is again in agreement with the previous results of cross-sectional research that support a relationship between increased fatigue and decreased productivity [29,30]. Of note, the association between fatigue index and productivity was only statistically significant in the fully adjusted model, which indicates that other explanatory demographic factors may contribute to this association. Given the correlation between job satisfaction and productivity as well as fatigue index and productivity, we considered the possibility of a mediating relationship, where these factors might serve as mediators between sedentary time and productivity. However, given that the correlation between sedentary time and productivity was not statistically significant, it would not be appropriate to conduct a mediation analysis using our data. Were such an analysis to be conducted, given the cross sectional design of the study, the true nature and direction of the mediation would be inscrutable. While not the main focus of this study, statistical adjustment according to multiple demographic factors indicated that age, gender, and education level were significantly associated with productivity (see Table 4). Overall, the current study confirms the importance of monitoring fatigue when examining workplace productivity. Follow-on analyses indicated that those in the lowest level of time spent sitting reported significantly lower fatigue overall as compared with those in the highest level of time spent sitting. This is consistent with previous evaluations of worksite-based interventions that have demonstrated reductions in sedentary behavior and self-reported fatigue [31,32]. Overall, the unadjusted analysis indicates a potential positive effect of reduced sitting time on fatigue in office workers, however, causality cannot be inferred. Longitudinal studies that help to elucidate directional relationships between sitting, fatigue, and productivity are needed to shed more light on potential mechanisms through which sitting exerts effects.

### 4.1. Limitations

There are several limitations to consider when interpreting the results of the current study. The primary limitation is the self-report measures of sedentary behavior and productivity. Although Chau and colleagues [17] reported a moderate-to-strong correlation (*r* = 0.65) between objectively measured sitting time and self-reported sitting time, estimates of sitting time do not capture actual sitting time precisely. Similarly, self-reports of productivity may not reflect actual productivity. Previous research using the Health and Work Questionnaire (HWQ) has suggested a significant association between self-reported productivity estimates and one objective measure of productivity, total hours lost (time unavailable to accept telephone calls without an authorized excuse), but the correlation was low (*r* = −0.195) [34]. In the same study, however, a more comprehensive objective assessment of productivity, total performance points, was not significantly associated with self-reported productivity. Productivity is challenging to measure objectively; it requires measures to be tailored to the specific responsibilities of the employee sample [40]. Objective productivity measures also limit the ability for studies to include samples with varying occupational duties. Thus, the current study design was appropriate for an easily captured estimate of productivity across a large sample of participants who did not share the same work responsibilities.

Though this cross-sectional study demonstrates a number of significant associations, longitudinal investigations of these factors are needed to determine causal relationships. Another limitation is that the study sample may not be representative of the specific worksites included in the study. This survey was administered as part of an interest survey for a larger study of sedentary behavior in the workplace. Although all employees were encouraged to complete the survey, it is possible that the sample of employees who were willing to participate in the interest survey was not representative of the population of interest for this study; that is, employees of large state government agencies in the State of Kansas. Furthermore, this study focused on only one work sector. The findings of this study may not be generalizable to office workers within other work sectors. Finally, this study used selected items from full questionnaires to measure fatigue, productivity, and job satisfaction. The selections were made in a way that maintained subscales could be analyzed in a similar or identical fashion to the original scales, as evidenced by the high internal consistency values for productivity (α = 0.88) and job satisfaction (α = 0.84). However, there are additional items within these scales that could have provided a more comprehensive assessment of these three constructs. Ultimately, the survey length was appropriate for the purpose of identifying interest in participation; it allowed for the opportunity to gain some initial insight into the relationships of interest from a large sample of primarily sedentary office workers without an excessive time burden for participants.

### 4.2. Future Research

Researchers can build on the findings of the current study by continuing to investigate the potential relationship between workplace sedentary behavior and productivity using both self-report and objective measures. In particular, studies that examine associations between fatigue and productivity are needed in order to answer questions related to the potential for reduced sitting to lead to reductions in fatigue that may enhance job satisfaction and productivity. It is important to determine whether sitting and fatigue are part of a feedback loop linking sedentary behavior to fatigue, leading to reductions in productivity. Studies should seek to understand how other factors such as age and gender play a role in actual and perceived productivity.

The absence of a significant relationship between sitting time and productivity within the current study may also suggest that additional measures of related constructs are necessary to understand the relationships between workplace sedentary behavior and work-related outcomes. For instance, there may be differences in productivity between those who engage in light versus moderate physical activity at work when they have similar amounts of accumulated sedentary time [16]. Moreover, measures of other work-related outcomes (e.g., focus) could help illustrate the effects of sedentary behavior in the workplace [32]. Additional evidence related to each of these potential relationships could produce a more comprehensive understanding of workplace sedentary behavior and productivity.

## 5. Conclusions

The current study provides an initial assessment of workplace sedentary behavior among full-time state government office workers, and its relationship with productivity. The data support previous studies that demonstrate the pervasiveness of sedentary behavior in the workplace, which is a cause for concern regarding health. Overall, the results suggest that sitting time at work is not associated with productivity, and office workers in the lowest level of time spent sitting report higher job satisfaction and lower fatigue as compared with those in the highest level of time spent sitting. Future investigations of sedentary behavior and productivity will require a more comprehensive assessment of job satisfaction, fatigue, and other related factors, using more objective methods where possible, in order to provide meaningful contributions to better understand workplace sedentary behavior and associations with work-related outcomes. For the time being, as researchers and public health professionals engage with employers to promote reductions in sedentary behavior in office workers, these data offer promising support that less sitting time is associated with positive outcomes that do not seem to come at the expense of productivity.

## Figures and Tables

**Table 1:** Sample survey items.

Construct	Sample Item	Previous Source
Sedentary Behavior	Describe your typical work day in the last 7 days: % of time spent sitting (do not include driving)	Occupational Sitting and Physical Activity Questionnaire, Chau et al., 2012
Demographics	Are you in your office at least 30 hours per week (not including telecommuting)?	n/a
Productivity	How would you describe the OVERALL QUALITY of your work in the past 7 days?	Health & Work Questionnaire, Shikiar et al., 2004
Fatigue	Rate your level of fatigue on the average during the past week.	Fatigue Symptom Inventory, Hann et al., 1998
Job Satisfaction	How personally rewarding did you find your work in the past 7 days?	Health & Work Questionnaire, Shikiar et al., 2004

**Table 2:** Participant demographics.

Demographic Variable	Frequency	Percentage (%)
**Gender**		
Female	1476	71.9
Male	576	28.1
Sum	2052	100
**Race**		
White	1871	92.7
Black or African-American American	54	2.7
American Indian or Alaska Native	18	0.9
Asian American or Pacific Islander	13	0.6
Other race	24	1.2
Multiracial	39	1.9
Sum	2019	100
**Ethnicity**		
Hispanic/Latino/Spanish	160	7.9
Non-Hispanic/Latino/Spanish	1872	92.1
sum	2032	100
**Marital Status**		
Married	1261	61.6
Widowed	41	2.0
Divorced	280	13.7
Separated	20	1.0
Single	444	21.7
Sum	2046	100
**Education**		
Less than 9th grade	0	0
9th to 12th grade, no diploma	1	<1
High school diploma or GED	160	7.8
Some college, no degree	452	22.1
Associate degree	212	10.3
Bachelor’s degree	853	41.6
Graduate or professional degree	371	18.1
Sum	2049	100

Note: GED is a General Equivalency Diploma which is a high school equivalency diploma.

**Table 3:** Mean results for sedentary behavior, productivity, job satisfaction, and fatigue.

Occupational Variable	Scale ^#^	*M*	*SD*
**Sedentary Behavior**			
Percentage of Worktime Spent Sitting	0–100%	78.1	18.9
**Productivity**			
Overall Quality of Work	1–10 (Best ever)	7.8	1.4
Overall Amount of Work	1–10 (Best ever)	7.6	1.7
Work Efficiency	1–10 (Best ever)	7.3	1.6
Productivity Subscale Score		7.6	1.4
**Job Satisfaction**			
Satisfaction with Coworkers	1–10 (Very satisfied)	7.5	2.1
Overall Job Satisfaction	1–10 (Very satisfied)	6.6	2.2
Work is Personally Rewarding	1–10 (Very rewarding)	6.4	2.3
Satisfaction with Work Environment	1–10 (Very satisfied)	5.8	2.4
Job Satisfaction Subscale Score		6.6	1.8
**Fatigue**			
Average Fatigue Level	1–11 (As fatigued as I could be)	6.3	2.4
Amount of Day with Fatigue	1–11 (The entire day)	5.1	2.4
Number of Days with Fatigue	0–7	3.9	2.2
Overall Fatigue Index	0–847	179.7	184.4

Note: ^#^ parentheses indicate the meaning of the largest value.

**Table 4:** Associations between percent time spent sitting during a typical workday and productivity, statistically adjusting for job satisfaction, fatigue index, and demographic variables.

Variable	Standardized β	Adjusted R^2^	ΔR^2^	Significance
**Step 1**		0		0.48
Sitting time (%)	0.016	0.240	0.240	0.48
**Step 2**		0.24	0.24	<0.001
Sitting time (%)	−0.009			0.636
Job satisfaction	0.481			<0.001
Fatigue index	−0.036			0.086
**Step 3**		0.265	0.025	<0.001
Sitting time (%)	0.013			0.519
Job satisfaction	0.473			<0.001
Fatigue index	−0.047			0.023
Race (white, non-white)	−0.034			0.085
Age (years)	0.089			<0.001
Gender (male, female)	0.105			<0.001
Education level	−0.076			<0.001

## Appendix A. Survey Used for Data Collection

1. Select your current employer [dropdown list with government agencies]
2. Are you in your office at least 30 hours per week (not including telecommuting)?
  ☐ Yes
  ☐ No
3. How would you describe your typical work day in the last 7 days? (This involves only your work day, and does not include travel to and from work, or what you did in your leisure time)
  ☐ % of time spent sitting (do not include driving): ____
  ☐ % of time spent driving: ____
  ☐ % of time spent standing: ____
  ☐ % of time spent walking: ____
  ☐ % of time spent doing heavy labor or physically demanding tasks: ____
  ☐ % of time doing other activities: ____
  ☐ [total percentages had to total 100%]
4. How personally rewarding did you find your work in the past 7 days?

**1**									**10**
**Not rewarding at all**	**2**	**3**	**4**	**5**	**6**	**7**	**8**	**9**	**Very rewarding**

5. How satisfied were you in the past 7 days with the physical environment in which you work (e.g., amount of noise, temperature where you work)?

**1**									**10**
**Very Dissatisfied**	**2**	**3**	**4**	**5**	**6**	**7**	**8**	**9**	**Very Satisfied**

6. How satisfied were you overall with your job in the past 7 days?

**1**									**10**
**Very Dissatisfied**	**2**	**3**	**4**	**5**	**6**	**7**	**8**	**9**	**Very Satisfied**

7. How satisfied were you in the past 7 days with your relationships with your coworkers?

**1**									**10**
**Very Dissatisfied**	**2**	**3**	**4**	**5**	**6**	**7**	**8**	**9**	**Very Satisfied**

8. How would you describe your EFFICIENCY in the past 7 days?

**1**									**10**
**Very Dissatisfied**	**2**	**3**	**4**	**5**	**6**	**7**	**8**	**9**	**Very Satisfied**

9. How would you describe the OVERALL QUALITY of your work in the past 7 days?

**1**									**10**
**My worst ever**	**2**	**3**	**4**	**5**	**6**	**7**	**8**	**9**	**My best ever**

10. How would you describe the OVERALL AMOUNT of work you did in the past 7 days?

**1**									**10**
**My worst ever**	**2**	**3**	**4**	**5**	**6**	**7**	**8**	**9**	**My best ever**

11. Rate your level of fatigue on the average during the past week.

**0**										**10**
**Not at all fatigued**	**1**	**2**	**3**	**4**	**5**	**6**	**7**	**8**	**9**	**As fatigued as I could be**

12. Indicate how many days, in the past week, you felt fatigued for any part of the day.

**0**							**7**
**Days**	**1**	**2**	**3**	**4**	**5**	**6**	**Days**

13. Rate how much of the day, on average, you felt fatigued in the past week.

**0**										**10**
**None of the day**	**1**	**2**	**3**	**4**	**5**	**6**	**7**	**8**	**9**	**The entire day**

14. What is your gender?
  ☐ Male
  ☐ Female
15. What is your age? [entry box for age]
16. What is your marital status?
  ☐ Married
  ☐ Widowed
  ☐ Divorced
  ☐ Separated
  ☐ Single
17. What is your highest level of education completed?
  ☐ Less than 9th grade
  ☐ 9th to 12th grade, no diploma
  ☐ High school diploma or GED
  ☐ Some college, no degree
  ☐ Associate degree
  ☐ Bachelor’s degree
  ☐ Graduate or professional degree
18. Are you of Hispanic, Latino, or Spanish origin?
  ☐ Yes
  ☐ No
19. What is your race? (check all that apply)
  ☐ White
  ☐ Black or African American
  ☐ American Indian or Alaska Native
  ☐ Asian American or Pacific Islander
  ☐ Other (please specify)

## Appendix B. Table of Missing Value Imputations

	**Number of Missing Values**	**Percentage of Total Values**
**Productivity**		
How would you describe your EFFICIENCY in the past 7 days?	4	0.19
How would you describe the OVERALL QUALITY of your work in the past 7 days?	16	0.77
How would you describe the OVERALL AMOUNT of work you did in the past 7 days?	12	0.58
**Job Satisfaction**		
How personally rewarding did you find your work in the past 7 days?	4	0.19
How satisfied were you in the past 7 days with the physical environment in which you work (e.g., amount of noise, temperature where you work)?	3	0.14
How satisfied were you overall with your job in the past 7 days?	62	3.00
How satisfied were you in the past 7 days with your relationships with your coworkers?	44	2.13
**Total Missing Values (to be imputed)**	145	
